# Effects of Serious Games on Depression in Older Adults: Systematic Review and Meta-analysis of Randomized Controlled Trials

**DOI:** 10.2196/37753

**Published:** 2022-09-06

**Authors:** Yesol Kim, Soomin Hong, Mona Choi

**Affiliations:** 1 College of Nursing and Brain Korea 21 FOUR Project Yonsei University Seoul Republic of Korea; 2 Mo-Im Kim Nursing Research Institute College of Nursing Yonsei University Seoul Republic of Korea; 3 Yonsei Evidence Based Nursing Centre of Korea A JBI Affiliated Group Seoul Republic of Korea

**Keywords:** effectiveness, serious game, exergaming, video games, virtual reality, depression, older adults, systematic review, meta-analysis, mobile phone

## Abstract

**Background:**

Depression is a severe psychological concern that negatively affects health in older adults. Serious games applied in various fields are considered appropriate interventions, especially in mental health care. However, there is a lack of evidence regarding the effects of serious games on depression in older adults.

**Objective:**

This study aimed to investigate the characteristics and effectiveness of serious games for depression in older adults.

**Methods:**

A systematic review and meta-analysis of randomized controlled trials were conducted. In total, 5 electronic databases (PubMed, CINAHL, Embase, PsycINFO, and Cochrane Library) were searched to identify relevant studies published until July 6, 2021. A total of 2 reviewers independently conducted study selection, data extraction, and quality appraisals. The risk of bias in the included studies was assessed using the JBI Critical Appraisal Checklist. For the meta-analysis, the effect size was calculated as the standardized mean difference (SMD) by using a random effects model.

**Results:**

A total of 17 studies with 1280 older adults were included in the systematic review, and 15 studies were included in the meta-analysis. Serious game interventions were classified into 3 types: physical activity (PA), cognitive function, and both PA and cognitive function. The meta-analysis demonstrated that serious games reduced depression in older adults (SMD −0.54, 95% CI −0.79 to −0.29; *P*<.001). Serious games had a more significant effect size in community or home settings (SMD −0.61, 95% CI −0.95 to −0.26; *P*<.001) than in hospital settings (SMD −0.46, 95% CI −0.85 to −0.08; *P*=.02); however, the difference between groups was not significant. Among the types of games, games for PA (SMD −0.60, 95% CI −0.95 to −0.25; *P*<.001) and games for both (SMD −0.73, 95% CI −1.29 to −0.17; *P*=.01) had a significant effect on reducing depression in older adults. However, no significant correlations were observed between the duration or number of serious games and depression.

**Conclusions:**

Serious games were beneficial in reducing depression in older adults. Regardless of the study setting, serious games appeared to reduce depression. Particularly, serious games including PA had a significant impact on reducing depression. Furthermore, high-quality randomized controlled trials are needed to establish substantial evidence for the effectiveness of serious games on depression in older adults.

**Trial Registration:**

PROSPERO CRD42021242573; https://tinyurl.com/26xf7ym5

## Introduction

### Background

The aging population is increasing worldwide. The average life span has increased, and health-related concerns in older adults require attention [1], highlighting their physical and mental health concerns [2]. Researchers have examined the psychosocial aspects of older adults, including anxiety, depression, sleep disorders, loneliness, and social functional impairment [3-5]. Specifically, depression is a severe and typical mental health concern characterized by sadness and hopelessness [6]. Older adults are more vulnerable to depression owing to their psychosocial concerns. During the COVID-19 pandemic, older adults had a high risk of depression owing to a decrease in social relations, regardless of their environment or context [7,8].

The clinical conditions of older adults also make them likely to develop depression. Physical and cognitive problems and functional loss are the primary causes of depression in older adults [2]. Older adults experience ambiguous symptom profiles of depression, and atypical symptoms make early detection challenging [9]. In addition, insufficient psychosocial relationships and decreased economic status after retirement lead to depression in older adults [10,11].

The prevention, early detection, and treatment of depression in older adults are crucial. However, older adults tend to avoid using mental health services because of poor physical function, psychological barriers, and reduced mobility [12]. To improve the mental health of older adults, specific methods are required that consider their characteristics and attributes. Moreover, detailed and personalized interventions are required to manage depression in older adults. For instance, physical function, cognitive function (CF), sensory function impairment, comorbidities, medication, and environmental factors should be considered [13-16].

Digital interventions for mental health care are considered promising [17,18] and have become indispensable since the COVID-19 pandemic. Digital interventions have been found to be effective in reducing the symptoms of depression [19], loneliness [5], and social isolation [20] in older adults. There has been a gradual increase in the use of digital interventions for older adults in clinics and research, although digital interventions may pose certain challenges to older adults [21,22]. A mixed methods study has identified that although older adults may wish to make use of digital interventions to alleviate depression, they might also initially face certain obstacles to participation [23]. Digital interventions that consider the daily lives of older adults, ease of use, and low cost may help reduce depression [23]. In light of their perspectives, circumstances, and contexts, it is necessary to develop and implement effective interventions for older adults.

Serious games, a type of digital intervention, refer to a series of activities performed by combining the aspects of video games for specific purposes, such as education or rehabilitation [24]. Initially, they were developed for military purposes in the 1970s and recently appeared in more advanced forms with the development of computers and mobile devices [25]. In addition, they are now widely used in education and health care [26], such as physical rehabilitation [27], cognitive training [28,29], and health promotion [30]. Interest in serious games which allow participants to voluntarily achieve their goals has increased.

Various interventions of serious games have been conducted not only for adolescents [31] and younger adults [32] but also for older adults [33-35]. They were applied to older adults in different ways, including video games, using devices such as Nintendo [36,37], and virtual reality serious games [38]. When developing serious game interventions to improve the health of older adults, there are a variety of goals, such as strengthening physical function or CF. In addition, the composition or content of serious games was altered to fit the purpose of the intervention. For instance, if a serious game is designed to improve physical function, older adults require to move their bodies during the game [39]. In a previous study, a serious game was used and evaluated to enhance spatial memory in older adults by implementing the appearance of a real-world city and systematically applying a virtual environment [40].

Serious games are considered appropriate interventions in mental health care, including for the general population and patients with or without psychiatric concerns [41-43]. Studies have reported that serious games influence depression [44,45]; however, a systematic review and meta-analysis focusing on the effect size of a serious game on depression in older adults is rare. In addition, the intervention effects differed in studies on serious games for older adults [33,46-48]. Therefore, further analysis of serious games for depression in older adults is required.

### Aims

This systematic review and meta-analysis aimed to analyze the effects of various types of serious games on depression in older adults. The detailed research questions leading to this study are as follows: (1) What are the characteristics of serious games used to intervene in depression among older adults? (2) How effective are serious games to intervene in depression among older adults? (3) Which aspects of serious games affect depression in older adults?

## Methods

### Design

This systematic review and meta-analysis of randomized controlled trials were reported following the guidelines of PRISMA (Preferred Reporting Items for Systematic Reviews and Meta-Analyses) [49] and the JBI manual [50]. The study protocol was registered with PROSPERO (registration number CRD42021242573).

### Search Strategy

We conducted a systematic search by using electronic databases (PubMed, CINAHL, Embase, PsycINFO, and the Cochrane Library) on July 6, 2021. To organize the search terms, free text and Medical Subject Headings terms were combined according to the participants, interventions, comparisons, and outcomes. Key search terms were as follows: (“aged” or “older” or “elder*” or “senior”) and (“game” or “gaming” or “exergame” or “serious game” or “serious gaming”) and (“depression” or “depressive disorder”). A summary of search strategies is presented in Multimedia Appendix 1.

### Eligibility Criteria

Eligibility criteria were determined according to the participants, interventions, comparisons, outcomes, and study design. The inclusion criteria were as follows: (1) participants—studies that included older adults with a mean age of ≥65 years; (2) interventions—studies that applied serious games comprising exergames, virtual reality games, or digital games; (3) comparisons—studies that applied usual care or nonserious games for the control group; (4) outcomes—studies measuring depression; and (5) study design—randomized controlled trials only. The exclusion criteria were as follows: (1) studies that applied different doses or intensities of the serious game for the control group; (2) studies published in a language other than English; and (3) gray literature such as theses, dissertations, or conference abstracts.

### Study Selection

After searching for studies in electronic databases, one researcher (YK) exported all the studies to the reference management software EndNote X9 (Clarivate Analytics), and the other researchers (SH and MC) rechecked the extracted studies. The titles and abstracts of the extracted studies were independently screened according to the inclusion criteria by 2 researchers (YK and SH). Subsequently, they reviewed the full texts separately to select the final studies to be included. If the screening results did not match, a consensus was reached through discussion. The other researcher (MC) supervised the screening process.

### Data Extraction

Two researchers (YK and SH) independently extracted the data. The data extraction of the selected studies was performed using a structured form that included study, participant, intervention, and outcome characteristics. First, the study characteristics included authors, publication year, country, and setting. Second, the participant characteristics included health status, age (mean and SD), and sample size. Intervention characteristics consisted of type, the device used, content, duration, frequency, time, dose of serious games, type of control group, and the interventionist. Finally, outcome characteristics comprised the measurement of depression, the main result, mean, and SD for the experimental and control groups. If data required for analysis could not be found in the article, researchers requested data from the respective authors via email.

### Risk-of-Bias Assessment

Two researchers (YK and SH) independently evaluated the methodological quality of the included studies according to the JBI Critical Appraisal Checklist for randomized controlled trials [50]. The checklist has 13 items to assess the risk of bias, including participants, assignments, measurement, and analysis domains, and 1 overall appraisal item. After each included study was assessed using a 13-item checklist as “yes,” “no,” and “unclear,” the final quality judgment was drawn according to the “yes” ratio. The risk of bias was evaluated as follows: (1) ≥75% was ranked as high quality, (2) the range between 50% and 74% was ranked as medium quality, and (3) <50% was considered poor quality [51].

### Data Analysis

We calculated effect sizes as the standardized mean difference (SMD) with a 95% CI by using the mean and SD to synthesize the pooled effect of serious games on depression in older adults. When depression values were not presented as mean and SD in original studies, SMD was calculated through the conversion process by using SE, median, range, or IQR [52,53]. On the basis of the study by Cohen [54], the effect sizes were considered small (0.2≤SMD<0.5), medium (0.5≤SMD<0.8), and large (SMD≥0.8). To identify the effects of heterogeneity in the meta-analysis, *I*^2^ was used. Heterogeneity was classified as low (25%), moderate (50%), or high (75%) according to *I*^2^ values [55]. Because the characteristics of the participants and interventions included in the meta-analysis were heterogeneous, we conducted a meta-analysis by using a random effects model. According to the heterogeneity results, a subgroup analysis was conducted on the setting, participant characteristics, type of serious game, and the control group. In addition, we performed a meta-regression to explore the causes of heterogeneity regarding the duration and dose of serious games. Publication bias was assessed using funnel plots and Egger test. Statistical significance was set at *P*<.05. Statistical analysis was performed using the Comprehensive Meta-analysis software (version 3.0) and Review Manager software (version 5.4).

## Results

### Study Selection

**Figure 1:**
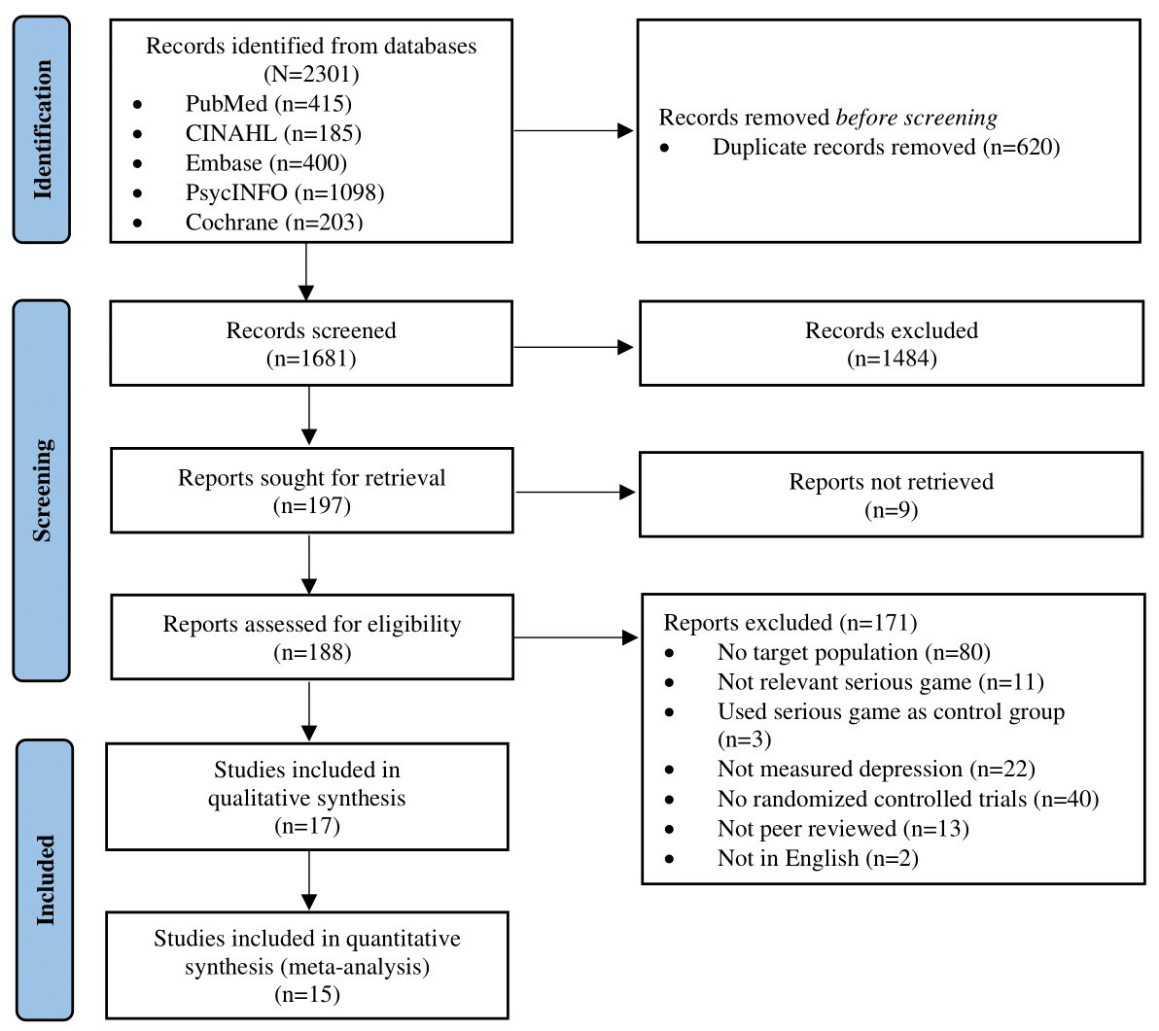
PRISMA (Preferred Reporting Items for Systematic Reviews and Meta-Analyses) 2020 flow diagram for study screening and selection.

Figure 1 shows a PRISMA 2020 flow diagram for screening and selection. A total of 2301 studies were extracted from 5 databases. After removing duplicate records (n=620, 26.94%), 1681 (73.06%) studies were screened using the inclusion and exclusion criteria based on title, abstract, and full text. Finally, 0.74% (17/2301) of studies were included in this study.

### Risk of Bias of the Studies

The results for the risk of bias in this study are presented in Multimedia Appendix 2 [50-66]. As confirmed by the number that met the criteria “yes,” the distribution of satisfying the risk-of-bias evaluation items ranged from 4 (31%) to 10 (77%) out of 13 items. Only 1 study [56] was evaluated as high quality, which indicated a low risk of bias. In total, 5 studies were ranked as poor quality (<50%) [57-61]. The remaining 11 studies were categorized as medium quality, with a distribution of 54% to 69%.

### Study Characteristics

The characteristics of the selected studies are summarized in Table 1. A total of 17 studies were published between 2012 and 2021 and conducted in 10 countries. The United States had the most research conducted [57,58,60,62], followed by Brazil [61,63,64], Korea [59,65], and Hungary [66,67]. More than half of the studies were conducted in community settings such as retirement villages, nursing homes, long-term care facilities, and assisted living facilities [56,60-63,68-71]. In total, 5 studies were conducted in hospitals [59,64-67] and 3 in homes [57,58,72].

A total of 1280 older adults were included in this review. Among the 17 studies, 7 (41%) mentioned health problems as the criteria for participant selection. The health status characteristics of the participants included neurocognitive problems, such as Parkinson disease and predementia [64-66,71], depression [57], limited mobility [67], and stroke [59]. For the 3-arm study, control groups were indicated by “control group a” and “control group b” [58,64,66,67]. The mean age of the participants ranged from 66.4 (SD 0.8) [63] to 85.0 (SD 6.1) [71], and the sample size varied between 16 [69] and 351 [62].

Various measurements of depression in older adults were described in the included studies. In 35% (6/17) of the studies, the Geriatric Depression Scale, including 5, 15, and 30 items, was used to measure depression [58,60,64,65,68,70]. In 29% (5/17) of the studies, depression was measured using the Beck Depression Inventory [59,61,66,67,69]. Other studies (6/17, 35%) used the Patient Health Questionnaire-9 [56,62], Profile of Mood States [63,72], Cornell Scale for Depression in Dementia [71], and Hamilton Depression Rating Scale [57].

**Table 1:** Characteristics of the included studies (N=17).

Authors, year	Country, setting	Characteristics of participants	Age (years), mean (SD)	Sample size, n	Measure	Effect size^a^ (95% CI)
Rendon et al [60], 2012	United States, community	Community-dwelling older adults	E^b^: 85.7 (4.3); C^c^: 83.3 (6.2)	E: 20; C: 20	GDS^d^-30	−0.72 (−1.41 to −0.03)
Schoene et al [56], 2015	Australia, community	Community-dwelling older adults	81.5 (7.0)	E: 47; C: 43	PHQ-9^e^	−0.29 (−0.72 to 0.14)
Choi et al [59], 2016	Korea, hospital	Hospitalized patients with ischemic stroke	E: 61.0 (15.2); C: 72.1 (9.9)	E: 12; C: 12	BDI^f^	−0.11 (−0.91 to 0.69)
Levy et al [69], 2016	France, community	Community-dwelling older adults with the fear of falling	E: 72.4 (12.3); C: 68.7 (19.1)	E: 9; C: 7	BDI	−0.75 (−1.77 to 0.27)
Nouchi et al [72], 2016	Japan, home	Community-dwelling older adults	68.9 (3.7)	E: 36; C: 36	POMS^g^2	NC^h^
Anguera et al [57], 2017	United States, home	Major depression	68.0 (6.3)	E: 12; C: 10	HAM-D^i^	−0.13 (−0.97 to 0.71)
Ferraz et al [64], 2018	Brazil, hospital	Parkinson disease	69.0 (5.0)	E: 22; C-a^j^: 25; C-b^k^: 25	GDS-15	a: −0.10 (−0.71 to 0.51); b: −0.11 (−0.74 to 0.52)
Belchior et al [58], 2019	United States, home	Community-dwelling older adults	73.2 (5.5)	E: 26; C-a: 20; C-b: 25	GDS-30	NC
Smith et al [62], 2019	United States, community	Lived in supported senior living settings	80.6 (9.1)	E: 173; C: 178	PHQ-9	−0.10 (−0.34 to 0.14)
Stanmore et al [70], 2019	United Kingdom, community	Lived in assisted living facilities	E: 77.9 (8.9); C: 77.8 (10.2)	E: 56; C: 50	GDS-5	−0.17 (−0.58 to 0.24)
Tollár et al [66], 2019	Hungary, hospital	Parkinson disease	E: 70.0 (4.7); C-a: 70.6 (4.1); C-b: 67.5 (4.3)	E: 25; C-a: 25; C-b: 24	BDI	a: −0.17 (−0.72 to 0.38); b: −1.22 (−1.83 to −0.61)
Tollár et al [67], 2019	Hungary, hospital	Mobility-limited older adults	69.6 (3.5)	E: 28; C-a: 27; C-b: 28	BDI	a: −0.28 (−0.81 to 0.25); b: −1.46 (−2.05 to −0.87)
de Morais et al [63], 2020	Brazil, community	Older adults	66.4 (0.8)	E: 29; C: 29	POMS	−0.29 (−0.80 to 0.22)
Rica et al [61], 2020	Brazil, community	Institutionalized older women aged >60 years	Not reported	E: 16; C: 34	BDI	−2.08 (−2.81 to −1.35)
Jahouh et al [68], 2021	Spain, community	Institutionalized in nursing home or attending day center	E: 85.1 (8.6); C: 83.3 (8.8)	E: 40; C: 40	GDS-15	−0.65 (−1.10 to −0.20)
Kang et al [65], 2021	Korea, hospital	Predementia state	74.5 (5.8)	E: 25; C: 20	GDS-30	−0.19 (−0.82 to 0.44)
Swinnen et al [71], 2021	Belgium, community	Older adults with neurocognitive disorder residing in long-term care facilities	E: 84.7 (5.6); C: 85.3 (6.5)	E: 28; C: 27	CSDD^l^	−1.38 (−2.03 to −0.73)

^a^Effect size was calculated as the standardized mean difference with a 95% CI.

^b^E: experimental group.

^c^C: control group.

^d^GDS: Geriatric Depression Scale.

^e^PHQ-9: Patient Health Questionnaire-9.

^f^BDI: Beck Depression Inventory.

^g^POMS: Profile of Mood State.

^h^NC: not calculated because required data were not provided.

^i^HAM-D: Hamilton Depression Rating Scale.

^j^C-a: a control group of the 3-arm study.

^k^C-b: the other control group of the 3-arm study.

^l^CSDD: Cornell Scale for Depression in Dementia.

### Characteristics of the Serious Game Intervention

The characteristics of the serious game intervention are presented in Tables 2 and 3. Serious game interventions were classified into 3 types: games for physical activity (PA; 9/17, 53%), games for CF (5/17, 29%), and games for both PA and CF (3/17, 18%).

Regarding games for PA, the devices used in the intervention were Microsoft Xbox 360 [61,63,64,66,67]; Nintendo [60]; Microsoft Kinect [70]; tablet computers and smartphones with a Bluetooth connection [59]; and V8 Head Mount Display, 3D electromagnetic sensor, and PlayStation 2 [69]. Studies using the Xbox 360 primarily provided various commercial games that involved the movement of the participant’s body. Studies using Nintendo had applied strength training, aerobics, and balance games of Wii Fit. A study applying Kinect provided 16 exergames targeting the lower or upper limbs by using the Medical Interactive Recovery Assistant digital platform [70]. Choi et al [59] reported that a game improved the mobility of the upper extremity through a mobile app and Bluetooth connection to smart devices. A study using the V8 Head Mount Display, part of a virtual reality game, provided video games that required movements of the participants’ bodies [69].

Regarding games for CF, devices included tablet computers [57,72], controllers [58], CDs or computers [62], and Oculus Lift CV1 and touch controllers [65]. Studies using tablet computers applied the developed cognitive training game to participants. Commercial games that can improve CF were provided based on controllers, CDs, or websites. As part of a virtual reality game, a study using the Oculus Lift CV1 and touch controllers applied games that consisted of multidomain cognitive tasks.

Regarding games for both PA and CF, the devices used were electronic step pads [56,71] and Nintendo [68]. Studies applying electronic step pads provided step training that promoted both PA and CF, wherein participants moved in various directions. Another study provided various games that involved PA and CF using Nintendo (Table 2).

Among the 17 studies, 1 (6%) study [63] did not report the duration of intervention, whereas 16 (94%) studies reported the duration of intervention to be between 2 weeks [59] and 12 months [62]. Among the studies, the most frequent durations of intervention were reported as 8 weeks [57,64,68,71] and 12 weeks [58,61,69,70]. The prescribed serious game intervention was conducted for 1 to 5 sessions per week and 15 to 60 minutes per session. The total dose provided to the participants ranged from 2 sessions [63] to 60 sessions [58].

Of the included studies, the control groups consisted of usual care [58,60,65-70], exercise [59,64,66,67], nonserious games [61,62,72], watching a film or music videos [63,71], or other programs such as providing a brochure [56], cognitive training [58], and problem-solving therapy [57].

Of the 17 studies, 9 (53%) reported interventionists. Physical or occupational therapists [59,60,64,66,67,70,71], a neuropsychologist [65], and an interprofessional team [57] provided interventions to the participants (Table 3).

**Table 2:** Summary of serious game interventions of the included studies (N=17).

Authors, year	Type of serious game; device	Contents
Rendon et al [60], 2012	PA^a^; Nintendo	Wii fit using the Wii Balance Board Balance games (lunges, single leg extensions, and twists)
Schoene et al [56], 2015	Both; electronic step pad	The interactive training system used stepping onto an electronic step pad to interact with a computer interface, and videogame technology was used to deliver the training tasks on standard home television screens Videogames (Stepper, StepMania, Trail-Stepping, and Tetris)
Choi et al [59], 2016	PA; tablet computer and smartphone with Bluetooth connection	The MoU-Rehab consisted of 4 mobile game apps All game apps were designed to improve strength, endurance, range of motion, control, speed, and accuracy of movement in the upper extremity
Levy et al [69], 2016	PA; V8 Head Mount Display, 3D electromagnetic sensor, and EyeToy interface for PlayStation 2	Participants played video games that required moving their bodies Games (wash a window and kung fu)
Nouchi et al [72], 2016	CF^b^; tablet computer	In total, 12 processing speed training games to function on the tablet computer All games required participants to detect, identify, discriminate, and localize targets as quickly as possible
Anguera et al [57], 2017	CF; tablet computer	Mobile iPad intervention called Project: EVO based on the video game called NeuroRacer This game involves guiding a character through an immersive environment while responding to select targets, with the design format being ideally entertaining
Ferraz et al [64], 2018	PA; Xbox 360	Exergames use full-body motion to allow the player to engage in a variety of mini games, all of which feature jump-in, jump-out multiplayer play Physical components involved in those games included strength and muscular endurance, cardiorespiratory fitness, postural balance, and executive function
Belchior et al [58], 2019	CF; videogame and controller	Crazy taxi is a driving game with key features that include rapid navigation through an urban environment, attending to speed, and roadway features Characteristics of this game were speed; elevated perceptual, cognitive, and motor loads; and having items of interest often presented at the periphery of the visual field and under divided attention conditions
Smith et al [62], 2019	CF; CDs or web using computer	Road Tour on CDs and Double Decision, a web-based version, were used Road Tour and Double Decision performed the same way Speed of processing training participants saw an object (either a car or truck) in the center of the monitor and a target (route 66 road sign) along with 7 rabbit distractor signs in a near-periphery orbit. Participants viewed the monitor image as quickly as they could while still correctly identifying the object and the target location
Stanmore et al [70], 2019	PA; Microsoft Kinect	Kinect tracks the user’s performance and records parameters Each participant was given a prescribed program of standardized exergames that suited the participant’s starting level of ability with tailored progression Individual exercise programs can be tailored using a choice of games for lower or upper limb exercises using 16 of Medical Interactive Recovery Assistant’s exergames (strength, balance, coordination, and flexibility exercises)
Tollár et al [66], 2019	PA; Xbox 360	Exergame was designed to improve postural control, gait mobility, gait stability, turning, and dynamic and static Exergame used the 3 visual feedback modules of the Xbox 360 core system, Kinect Adventures video game (Reflex Ridge, Space Pop, and Just Dance)
Tollár et al [67], 2019	PA; Xbox 360	Exergame was designed to improve postural control, gait mobility, gait stability, turning, and balance Exergame used 3 Xbox 360 modules (Reflex Ridge, Space Pop, and Just Dance)
de Morais et al [63], 2020	PA; Xbox 360	Xbox Kinect—“Your Shape Fitness evolved” (Zen-Develop it, Pump it, Wall Breacker, Kick it, Hurricane, and Stack in Up) The games are classified as easy, medium, or hard levels, and only the easy level was used
Rica et al [61], 2020	PA; Xbox 360	For Kinect-based exercise protocol, balance games were included Kinect Sports Ultimate Collection, Your Shape Fitness Evolved, Dance Central, and Nike + Kinect Training
Jahouh et al [68], 2021	Both; Nintendo	The intervention made up of different activities with the Nintendo Wii Fit video game console An aerobic-type game was used as a warm-up exercise The next game was played specifically to work on attention, concentration, and memory. In this game, a goalkeeper throws balls or bears from both the left and right sides. The participant was required to lean to either side to avoid all possible bears and head all possible balls; in other words, the participants had to swing on the same side of the ball or on the opposite side of the bear To end the session, the participants had to choose a game that they wanted to try or play
Kang et al [65], 2021	CF; Oculus Rift CV1 and Oculus touch controllers	Training was accompanied by game elements to increase the interest and motivation of the participants Games involving multidomain cognitive tasks to assess
Swinnen et al [71], 2021	Both; Dividat Senso	Dividat Senso consisted of a step training platform that was sensitive to pressure changes The sensors detected steps in 4 directions: left, right, top, and bottom The platform was connected via a USB cable to a computer and a frontal television screen on which the exergames were displayed Participants interacted with the game interface by pushing foot on 1 of the 4 different arrows The games trained cognitive abilities The device provided real-time visual, auditory, and somatosensory (vibrating platform) cues and feedback to enrich the game experience

^a^PA: physical activity.

^b^CF: cognitive function.

**Table 3:** Characteristics of serious game interventions of the included studies (N=17).

Authors, year	Type of serious game	Duration, frequency, time per session, dose	Control group	Interventionist
Rendon et al [60], 2012	PA^a^	6 weeks, 3 sessions per week, 35-45 minutes, 18 sessions	Usual care	Physical therapist
Schoene et al [56], 2015	Both	16 weeks, 3 sessions per week, 20 minutes, 48 sessions	Brochure	NR^b^
Choi et al [59], 2016	PA	2 weeks, 5 sessions per week, 60 minutes, 10 sessions	Exercise (conventional occupational therapy)	Occupational therapist
Levy et al [69], 2016	PA	12 weeks, 1 session per week, <40 minutes, 12 sessions	Usual care	NR
Nouchi et al [72], 2016	CF^c^	4 weeks, 5 sessions per week, 15 minutes, 20 sessions	Nonserious game (knowledge quiz)	NR
Anguera et al [57], 2017	CF	8 weeks, 5 sessions per week (biweekly), <20 minutes, 20 sessions	Problem-solving therapy	Interprofessional team (clinicians, care managers, and therapists)
Ferraz et al [64], 2018	PA	8 weeks, 3 sessions per week, 50 minutes, 24 sessions	Exercise (functional training); exercise (bicycle)	Physical therapist
Belchior et al [58], 2019	CF	12 weeks, 5 sessions per week, 60 minutes, 60 sessions	Cognitive training; usual care	NR
Smith et al [62], 2019	CF	12 months, NR, NR (600 minutes per 5-6 weeks), NR	Nonserious game (computerized crossword puzzles)	NR
Stanmore et al [70], 2019	PA	12 weeks, 3 sessions per week, NR, 36 sessions	Usual care	Physical therapist
Tollár et al [66], 2019	PA	5 weeks, 5 sessions per week, 60 minutes, 25 sessions	Exercise (stationary cycling); usual care	Physical therapist
Tollár et al [67], 2019	PA	5 weeks, 5 sessions per week, 60 minutes, 25 sessions	Exercise (stationary cycling); usual care	Physical therapist
de Morais et al [63], 2020	PA	NR, NR, 30 minutes, 2 sessions	Watching a film	NR
Rica et al [61], 2020	PA	12 weeks, 3 sessions per week, 60 minutes, 36 sessions	Nonserious game (board games)	NR
Jahouh et al [68], 2021	Both	8 weeks, 2-3 sessions per week, 40-45 minutes, 20 sessions	Usual care	NR
Kang et al [65], 2021	CF	4 weeks, 2 sessions per week, 20-30 minutes, 8 sessions	Usual care	Neuropsychologist
Swinnen et al [71], 2021	Both	8 weeks, 3 sessions per week, 15 minutes, 24 sessions	Watching music videos	Physical therapist

^a^PA: physical activity.

^b^NR: not reported.

^c^CF: cognitive function.

### Effects of Serious Games on Depression

**Figure 2:**
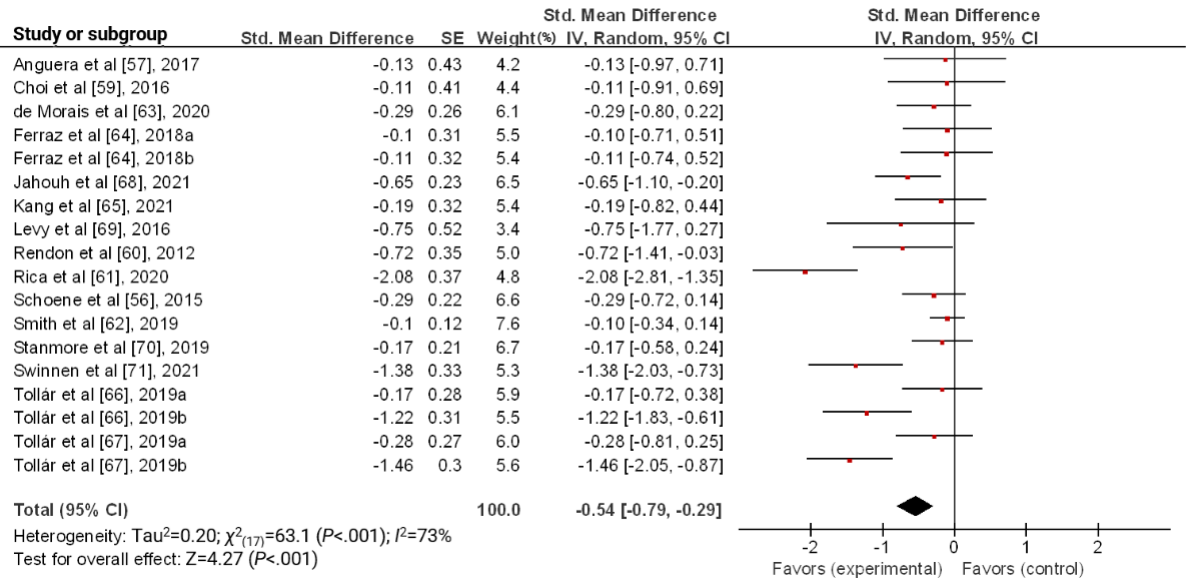
Forest plot for the effect of a serious game on depression.

Among the 17 studies included in the review, a meta-analysis was conducted on 15 (88%) studies, excluding 2 (12%) studies that did not provide raw data [58,72]. As 3 studies had 2 control groups each [64,66,67], we included 18 results in this meta-analysis. The pooled SMD between groups was −0.54 (95% CI −0.79 to −0.29; *P*<.001) with a medium effect size. These results indicate that serious games reduce depression in older adults. The heterogeneity of the meta-analysis was moderate to high across the studies (*I*^2^=73%; *P*<.001; Figure 2).

### Subgroup Analysis

**Figure 3:**
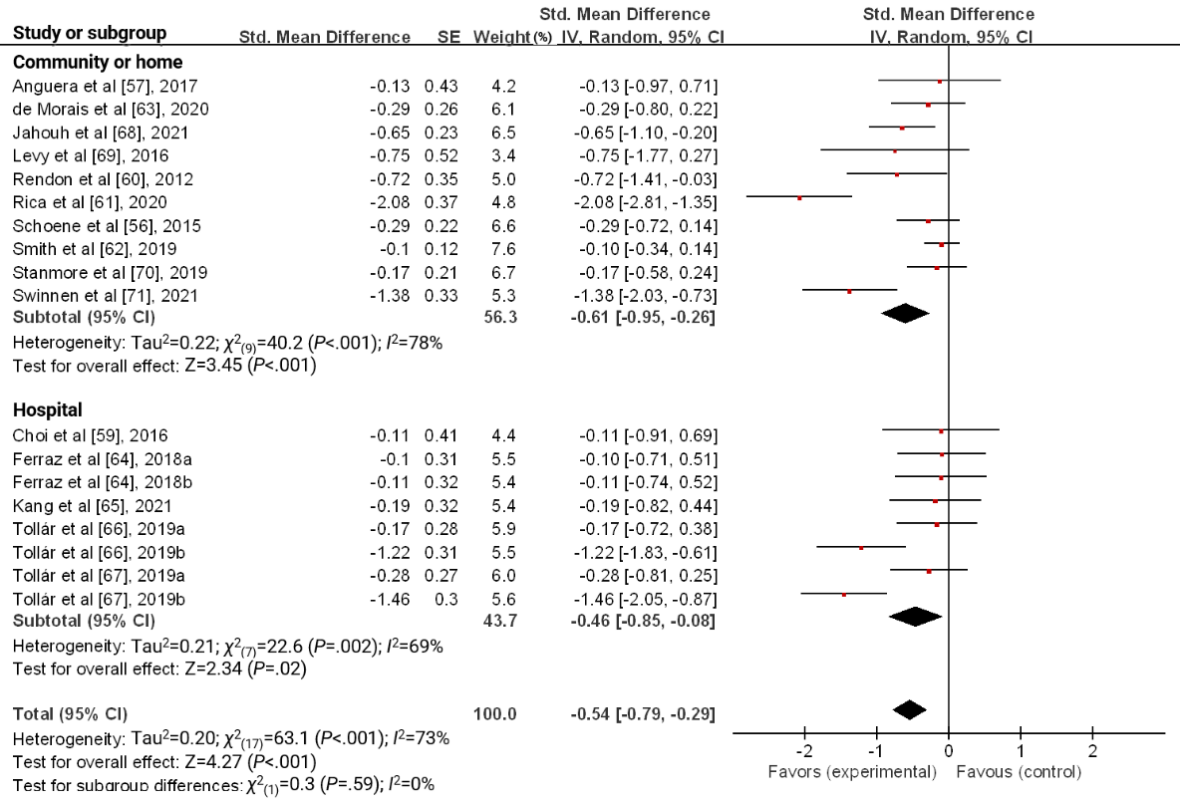
Forest plot for the effect of a serious game on depression according to setting.

**Figure 4:**
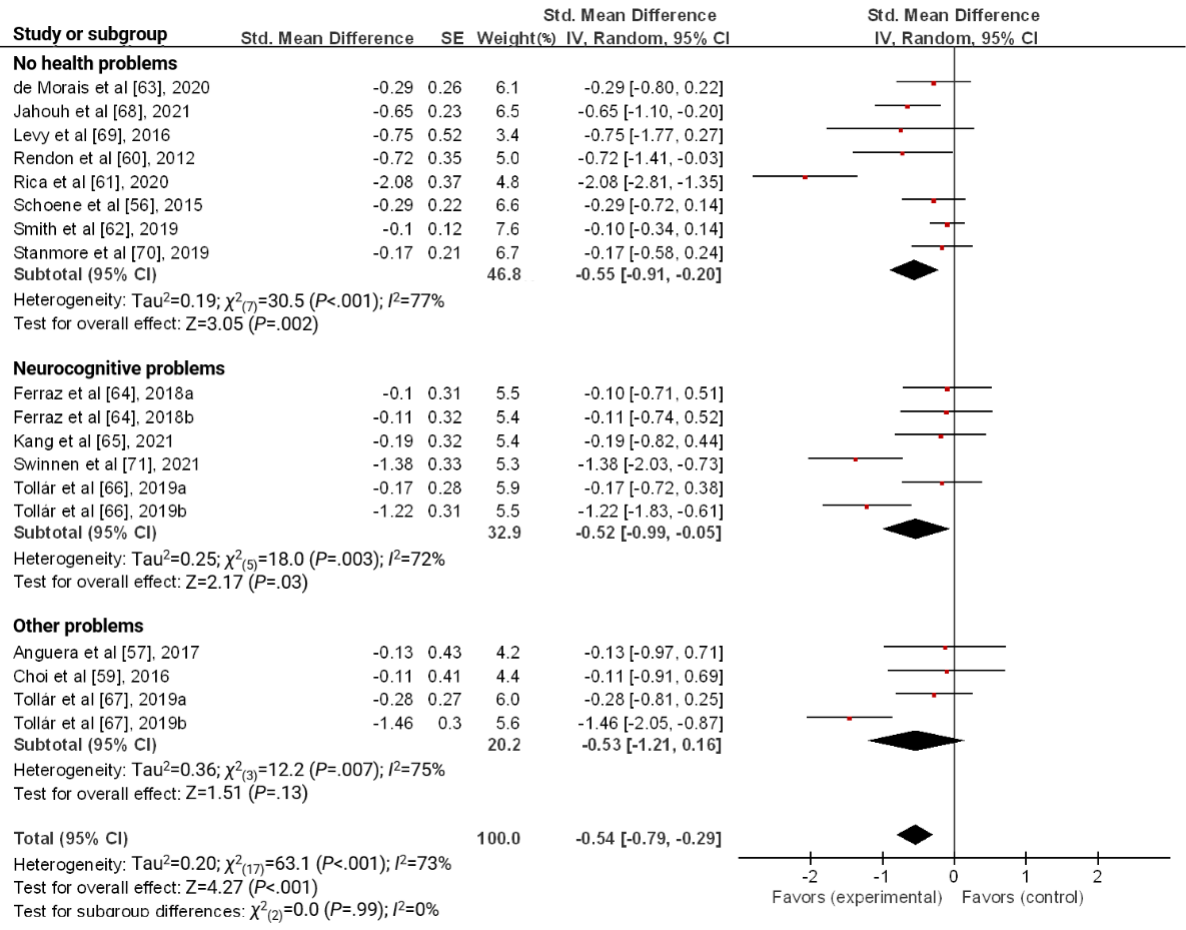
Forest plot for the effect of a serious game on depression according to the characteristics of participants.

**Figure 5:**
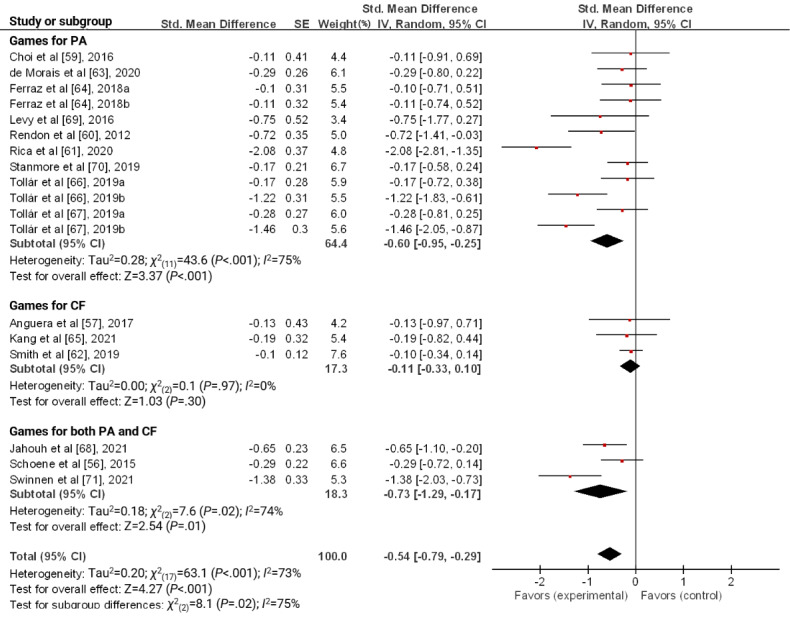
Forest plot for the effect of a serious game on depression according to the type of serious games. CF: cognitive function; PA: physical activity.

**Figure 6:**
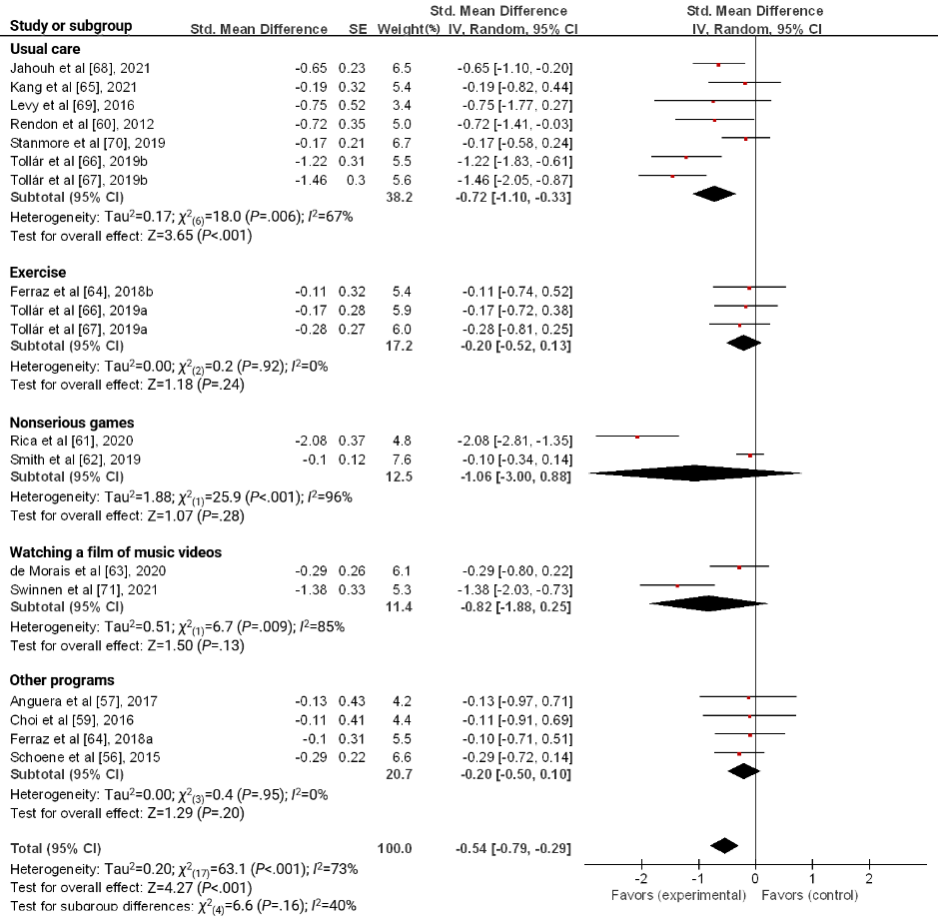
Forest plot for the effect of a serious game on depression according to the type of control group.

The results of the subgroup analysis of serious games for depression are shown in Figures 3-6. Regarding the setting, serious games had a more significant effect size in communities or homes (SMD −0.61, 95% CI −0.95 to −0.26; *P*<.001) than in hospitals (SMD −0.46, 95% CI −0.85 to −0.08; *P*=.02). However, the difference in the effect size between the groups was not statistically significant (*χ*^2^_1_=0.3; *P*=.59; Figure 3).

Regarding the characteristics of participants, the effect sizes of participants without health problems and with neurocognitive problems were −0.55 (95% CI −0.91 to −0.20; *P*=.002) and −0.52 (95% CI −0.99 to −0.05; *P*=.03), respectively, which significantly reduced depression (Figure 4).

A subgroup analysis of the type of serious game revealed that games for both had a significant effect on reducing depression in older adults (SMD −0.73, 95% CI −1.29 to −0.17; *P*=.01). In addition, games for PA significantly reduced depression (SMD −0.60, 95% CI −0.95 to −0.25; *P*<.001), whereas there was no significant effect of games on CF (Figure 5).

In the control group, serious games versus usual care had a significant effect on reducing depression (SMD −0.72, 95% CI −1.10 to −0.33; *P*<.001). However, subgroups of serious games versus other active comparators such as exercise, nonserious games, watching a film or music videos, and other programs presented no significant effect (Figure 6).

The results of the meta-regression indicated that no significant correlation existed between depression and the duration (*P*=.40) or dose of serious games (*P*=.43; Multimedia Appendix 3).

### Publication Bias

In this study, the publication bias was considered low. The funnel plot revealed a fairly symmetrical pattern (Multimedia Appendix 4). In addition, Egger test demonstrated that no publication bias was present in this meta-analysis (*P*=.27).

## Discussion

### Principal Findings

This study aimed to investigate the characteristics of serious games and their effects on depression in older adults. A total of 17 studies included in the systematic review have been conducted since 2012, and the number of studies has been steadily increasing. This study identified 3 types of serious games: games for PA, games for CF, and games for both PA and CF. Furthermore, it was demonstrated that the equipment and components of the game varied for each type of serious game. In addition, this study could provide substantial scientific evidence and produce high-quality findings as it included randomized controlled trials.

Older adults may face certain barriers to the use of digital interventions, such as physical changes because of aging and a lack of knowledge about technology [73]. In addition, studies included in this review reported that older adults who participated in serious games might experience difficulties with costs related to devices or programs [58], use of technology [70], and physical symptoms such as nausea, oculomotor dysfunction, and disorientation [65]. Despite these obstacles, the results of our meta-analysis indicate that serious games significantly reduce depression in older adults to a moderate effect size (SMD=−0.54). This aligns with the meta-analysis findings that showed reduced depression in young people [45]. In addition, the effect size of our findings was larger than that of studies targeting the general population [44,74]. Compared with the young or general population, serious games may effectively reduce depression in older adults, who face barriers in the application of digital interventions. As overcoming obstacles related to digital intervention may increase the interest and confidence of older adults [75], a serious game is considered effective and acceptable for them.

In this study, interventions of serious games were primarily conducted in places where older adults lived their daily lives, such as communities and homes, rather than in hospitals. Our findings showed that serious games played in communities or homes significantly reduced depression. Even without a supervised environment, such as a hospital, we found that the settings did not significantly affect older adults in applying serious games. This can be particularly convenient when applying the intervention to older adults with poor mobility or other accompanying diseases [47]. Therefore, a serious game can be applied regardless of the location once the appropriate environment or equipment is prepared.

In a subgroup analysis, serious games reduced depression in older adults without health problems or with neurocognitive problems. Appropriate physical abilities are required to perform serious games [76]; the intervention was effectively provided to older adults without health problems. Moreover, depression is considered an important health issue in older adults with neurocognitive problems such as Parkinson disease [77] and the predementia stage [78]. Accordingly, the findings of this study might be promising, as serious games may help reduce depression. However, the evidence may be relatively weak owing to the small number of studies. Further intervention studies are needed to confirm the relationship between the characteristics of older adults and the effectiveness of games.

Among the types of games, those that applied PA, including games for both accounted for approximately 70%. Our findings indicated that helping body movements directly by using various devices had a significant effect on reducing depression compared with games for CF. PA has been found to reduce depression through biological and psychosocial mechanisms [79]. In addition, a previous meta-analysis illustrated that exercise can significantly reduce depression in older adults [80]. Games promoting PA suggest the possibility of improving the quality of life and reducing depressive symptoms in older adults [61]. Therefore, PA should be considered as an essential component in the application of serious games to manage depression in older adults.

In the meta-analysis, we included studies that provided a control group with usual care as well as studies that provided other interventions considered active comparators. For studies comparing usual care groups, participation in serious games was found to have a significant impact on reducing depression. However, studies comparing active comparators, such as exercise, nonserious games, and watching videos, showed a reduction in depression, but this was not statistically significant. These findings are consistent with those of a meta-analysis that included active comparators [81]. The absolute and relative effects of the intervention can be interpreted according to the type of control group [82]. When the control group received usual care, the results indicated an absolute effect of the intervention. However, when the control group had active comparators, the results demonstrated a relative effect of the intervention. In this study, the absolute effect of participation in serious games was confirmed, but the relative effect was not. These findings indicate that participation in serious games has a unique and significant effect on older adults. Among the advantages of serious games, older adults can participate in such games regardless of the location [59], and it is generally easy to participate in such games [57]. In addition, participation in serious games motivates older adults and improves engagement [58,61], which has been found to increase adherence to interventions [57,63]. Participation in serious games for older adults may be viewed as an acceptable strategy to reduce depressive symptoms, as it has been confirmed that they have relatively high interest, satisfaction, and usability in serious games [59,65,70]. Therefore, we suggest that it is necessary to develop serious games that reflect helpful characteristics so that the relative effect as well as the absolute effect of interventions can be confirmed.

### Limitations

This study systematically reviewed the literature and analyzed the effectiveness of an overall serious game conducted to manage depression, without limiting the health characteristics of older adults. However, this study has a few limitations. First, some studies in which depression was not the primary outcome of the intervention were also selected because depression itself was of interest in this study. Generally, this selection may have a weak causal relationship between serious games and depression. In this review of 17 studies, 9 (53%) and 8 (47%) studies measured depression as a primary and secondary outcome, respectively. Depression had a significant reduction effect in 5 out of 9 (56%) studies measured as a primary outcome and 38% (3/8) of studies measured as a secondary outcome. The effects are greater when the intervention is performed for the main purpose of reducing depression. Therefore, further research is needed to clarify the relationship between depression as an outcome of intervention and its effectiveness. Second, methodological quality appraisals were performed using the JBI Critical Appraisal Checklist for the 17 studies included in this review. Only 6% (1/17) of high-quality studies had a low risk of bias, and approximately one-third (n=5, 29%) of the studies had a high risk of bias. Therefore, it can be considered that the evidence of the synthesized results is relatively low. Finally, this study included a variety of health characteristics, devices, and contents of serious games, which might lead to moderate to high heterogeneity (*I*^2^=73%). Thus, it is necessary to pay attention to the interpretation of the results.

### Implications

Our findings may contribute to the understanding of the effects of serious games on reducing depression in older adults. The findings of this study also provide researchers and health care providers with several implications for managing depression in older adults. First, there is a need to apply serious games involving PA to manage depression in older adults. It may be beneficial to add various types of serious games to increase their effectiveness. Second, serious games should be developed and adapted to suit the various characteristics and needs of older adults. In addition, it is necessary to further explore devices, content, and duration that will be effective for older adults. Finally, a large-scale and more rigorously designed randomized controlled trial of serious games should be conducted to provide scientific evidence.

### Conclusions

The findings of this review and meta-analysis demonstrate that serious games are beneficial for reducing depression in older adults. Among the types of serious games, those that include PA significantly reduce depression. Regarding the setting, interventions conducted in the community, including homes, can alleviate depression in older adults. We also found that studies provided by nurses or multidisciplinary teams were limited; therefore, nurse researchers should conduct serious game interventions further. In addition, more high-quality randomized controlled trials are needed to establish substantial evidence of the effectiveness of serious games on depression in older adults.

## Appendix

Multimedia Appendix 1 Search strategies and results of this study.

Multimedia Appendix 2 Methodological appraisal of the included studies (N=17).

Multimedia Appendix 3 Results of the meta-regression for subgroups of a serious game on depression.

Multimedia Appendix 4 Funnel plot of this study.

## Abbreviations

CF: cognitive function
PA: physical activity
PRISMA: Preferred Reporting Items for Systematic Reviews and Meta-Analyses
SMD: standardized mean difference
