# Supplementary material for: Effects of Serious Games on Depression in Older Adults: Systematic Review and Meta-analysis of Randomized Controlled Trials
Source: J Med Internet Res. 2022 Sep 6;24(9):e37753. doi: 10.2196/37753 (PMC9490522; doi:10.2196/37753)
Supplement: Multimedia Appendix 1 [file jmir_v24i9e37753_app1.docx]

## Search strategies and results of this study

(a) PubMed

| **Search number** | **Search terms** | **Search results** |
| --- | --- | --- |
| 1 | aged OR older OR elder* OR senior | 5891124 |
| 2 | "Aged"[Mesh] | 3270110 |
| 3 | #1 OR #2 | 5891124 |
| 4 | game OR gaming OR exergame OR "serious game" OR "serious gaming" | 47877 |
| 5 | "Video Games"[Mesh] OR "Games, Recreational"[Mesh] | 6439 |
| 6 | #4 OR #5 | 47877 |
| 7 | Depression OR "Depressive disorder" | 544266 |
| 8 | Depression[Mesh] OR Depressive Disorder[Mesh] OR Depressive Disorder, Major[Mesh] OR "Dysthymic Disorder"[Mesh] | 229877 |
| 9 | #7 OR #8 | 544266 |
| 10 | #3 AND #6 AND #9 | 415 |

(b) CINAHL

| **Search number** | **Search terms** | **Search results** |
| --- | --- | --- |
| 1 | aged OR older OR elder* OR senior | 1,127,740 |
| 2 | (MH "Aged+") | 877,368 |
| 3 | S1 OR S2 | 1,127,755 |
| 4 | game OR gaming OR exergame OR "serious game" OR "serious gaming" | 27,264 |
| 5 | (MH "Video Games") OR (MH "Exergames") | 5,154 |
| 6 | S4 OR S5 | 27,264 |
| 7 | Depression OR "Depressive disorder" | 182,606 |
| 8 | (MH "Depression") | 115,022 |
| 9 | S7 OR S8 | 182,606 |
| 10 | S3 AND S6 AND S9 | 185 |

(c) EMBASE

| **Search number** | **Search terms** | **Search results** |
| --- | --- | --- |
| 1 | aged OR older OR elder* OR senior | 5372261 |
| 2 | 'aged'/exp | 3314002 |
| 3 | #1 OR #2 | 5372261 |
| 4 | game OR gaming OR exergame OR 'serious game' OR 'serious gaming' | 44864 |
| 5 | 'video game'/exp OR 'recreational game'/exp | 4833 |
| 6 | #4 OR #5 | 44864 |
| 7 | depression OR 'depressive disorder' | 742349 |
| 8 | 'depression'/exp | 534638 |
| 9 | #7 OR #8 | 787977 |
| 10 | #3 AND #6 AND #9 | 400 |

(d) PsycINFO

| **Search number** | **Search terms** | **Search results** |
| --- | --- | --- |
| 1 | aged OR older OR elder* OR senior | 2187036 |
| 2 | MAINSUBJECT.EXACT("Older Adulthood") OR MAINSUBJECT.EXACT("Geriatric Patients") | 20864 |
| 3 | S1 OR S2 | 2187162 |
| 4 | game OR gaming OR exergame OR "serious game" OR "serious gaming" | 54502 |
| 5 | MAINSUBJECT.EXACT("Computer Games") OR MAINSUBJECT.EXACT("Digital Gaming") OR MAINSUBJECT.EXACT("Games") | 18510 |
| 6 | S4 OR S5 | 54502 |
| 7 | Depression OR "Depressive disorder" | 361219 |
| 8 | MAINSUBJECT.EXACT("Major Depression") OR MAINSUBJECT.EXACT("Depression (Emotion)") | 156262 |
| 9 | S7 OR S8 | 361219 |
| 10 | S3 AND S6 AND S9 | 1098 |

(e) Cochrane Library

| **Search number** | **Search terms** | **Search results** |
| --- | --- | --- |
| 1 | aged OR older OR elder* OR senior | 574955 |
| 2 | MeSH descriptor: [Aged] explode all trees | 212777 |
| 3 | #1 OR #2 | 574955 |
| 4 | game OR gaming OR exergame OR "serious game" OR "serious gaming" | 4708 |
| 5 | MeSH descriptor: [Video Games] explode all trees | 729 |
| 6 | MeSH descriptor: [Games, Recreational] explode all trees | 46 |
| 7 | #4 OR #5 OR #6 | 4923 |
| 8 | Depression OR "Depressive disorder" | 85053 |
| 9 | MeSH descriptor: [Depression] explode all trees | 12932 |
| 10 | MeSH descriptor: [Depressive Disorder] explode all trees | 12630 |
| 11 | MeSH descriptor: [Depressive Disorder, Major] explode all trees | 5196 |
| 12 | MeSH descriptor: [Dysthymic Disorder] explode all trees | 181 |
| 13 | #8 OR #9 OR #10 OR #11 OR #12 | 85130 |
| 14 | #3 AND #7 AND #13 | 203 |
