# Supplementary material for: Effects of Serious Games on Depression in Older Adults: Systematic Review and Meta-analysis of Randomized Controlled Trials
Source: J Med Internet Res. 2022 Sep 6;24(9):e37753. doi: 10.2196/37753 (PMC9490522; doi:10.2196/37753)
Supplement: Multimedia Appendix 2 [file jmir_v24i9e37753_app2.docx]

## Methodological appraisal of the included studies (N=17)

| Study | 1^a^ | 2^b^ | 3^c^ | 4^d^ | 5^e^ | 6^f^ | 7^g^ | 8^h^ | 9^i^ | 10^j^ | 11^k^ | 12^l^ | 13^m^ | Ratio of Y^n^ | Quality judgement |
| --- | --- | --- | --- | --- | --- | --- | --- | --- | --- | --- | --- | --- | --- | --- | --- |
| Rendon et al, 2012 [54] | Y | U^o^ | U | U | U | U | U | Y | Y | Y | U | Y | Y | 6/13  (46%) | Poor |
| Schoene et al, 2015 [50] | Y | Y | Y | Y | N^p^ | Y | U | Y | Y | Y | U | Y | Y | 10/13  (77%) | High |
| Choi et al, 2016 [53] | U | U | N | Y | N | Y | N | Y | U | Y | U | Y | Y | 6/13  (46%) | Poor |
| Levy et al, 2016 [63] | Y | U | Y | U | U | U | U | Y | Y | Y | U | Y | Y | 7/13  (54%) | Medium |
| Nouchi et al, 2016 [66] | Y | U | Y | Y | N | U | N | Y | Y | Y | U | Y | Y | 8/13  (62%) | Medium |
| Anguera et al, 2017 [51] | Y | U | Y | N | N | U | N | U | U | Y | Y | Y | Y | 6/13  (46%) | Poor |
| Ferraz et al, 2018 [58] | Y | Y | Y | N | N | Y | N | Y | U | Y | U | Y | Y | 8/13  (62%) | Medium |
| Belchior et al, 2019 [52] | U | U | U | N | N | Y | N | U | Y | Y | U | Y | Y | 5/13  (39%) | Poor |
| Smith et al, 2019 [56] | Y | Y | Y | Y | N | U | N | U | Y | Y | U | Y | Y | 8/13  (62%) | Medium |
| Stanmore et al, 2019 [64] | Y | N | Y | N | N | N | Y | Y | Y | Y | U | Y | Y | 8/13  (62%) | Medium |
| Tollár et al, 2019a [60] | Y | N | N | N | N | Y | Y | Y | U | Y | U | Y | Y | 7/13  (54%) | Medium |
| Tollár et al, 2019b [61] | U | U | Y | N | N | Y | Y | Y | U | Y | U | Y | Y | 7/13  (54%) | Medium |
| de Morais et al, 2020 [57] | U | U | Y | N | N | Y | Y | Y | U | Y | U | Y | Y | 7/13  (54%) | Medium |
| Rica et al, 2020 [55] | Y | U | U | N | N | U | N | U | U | Y | U | Y | Y | 4/13  (31%) | Poor |
| Jahouh et al, 2021 [62] | U | Y | Y | Y | N | Y | Y | Y | U | Y | U | Y | Y | 9/13  (69%) | Medium |
| Kang et al, 2021 [59] | Y | N | Y | N | N | Y | Y | Y | U | Y | U | Y | Y | 8/13  (62%) | Medium |
| Swinnen et al, 2021 [65] | Y | Y | Y | N | N | Y | N | Y | U | Y | U | Y | Y | 8/13  (62%) | Medium |

^a^Was true randomization used for assignment of participants to treatment groups?

^b^Was allocation to treatment groups concealed?

^c^Were treatment groups similar at the baseline?

^d^Were participants blind to treatment assignment?

^e^Were those delivering treatment blind to treatment assignment?

^f^Were outcomes assessors blind to treatment assignment?

^g^Were treatment groups treated identically other than the intervention of interest?

^h^Was follow up complete and if not, were differences between groups in terms of their follow up adequately described and analyzed?

^i^Were participants analyzed in the groups to which they were randomized?

^j^Were outcomes measured in the same way for treatment groups?

^k^Were outcomes measured in a reliable way?

^l^Was appropriate statistical analysis used?

^m^Was the trial design appropriate, and any deviations from the standard RCT design (individual randomization, parallel groups) accounted for in the conduct and analysis of the trial?

^n^Y: Yes.

^o^U: Unclear.

^p^N: No.
