# Supplementary material for: Effects of Serious Games on Depression in Older Adults: Systematic Review and Meta-analysis of Randomized Controlled Trials
Source: J Med Internet Res. 2022 Sep 6;24(9):e37753. doi: 10.2196/37753 (PMC9490522; doi:10.2196/37753)

## Results of the meta-regression for subgroups of a serious game on depression

(a) Duration of serious games


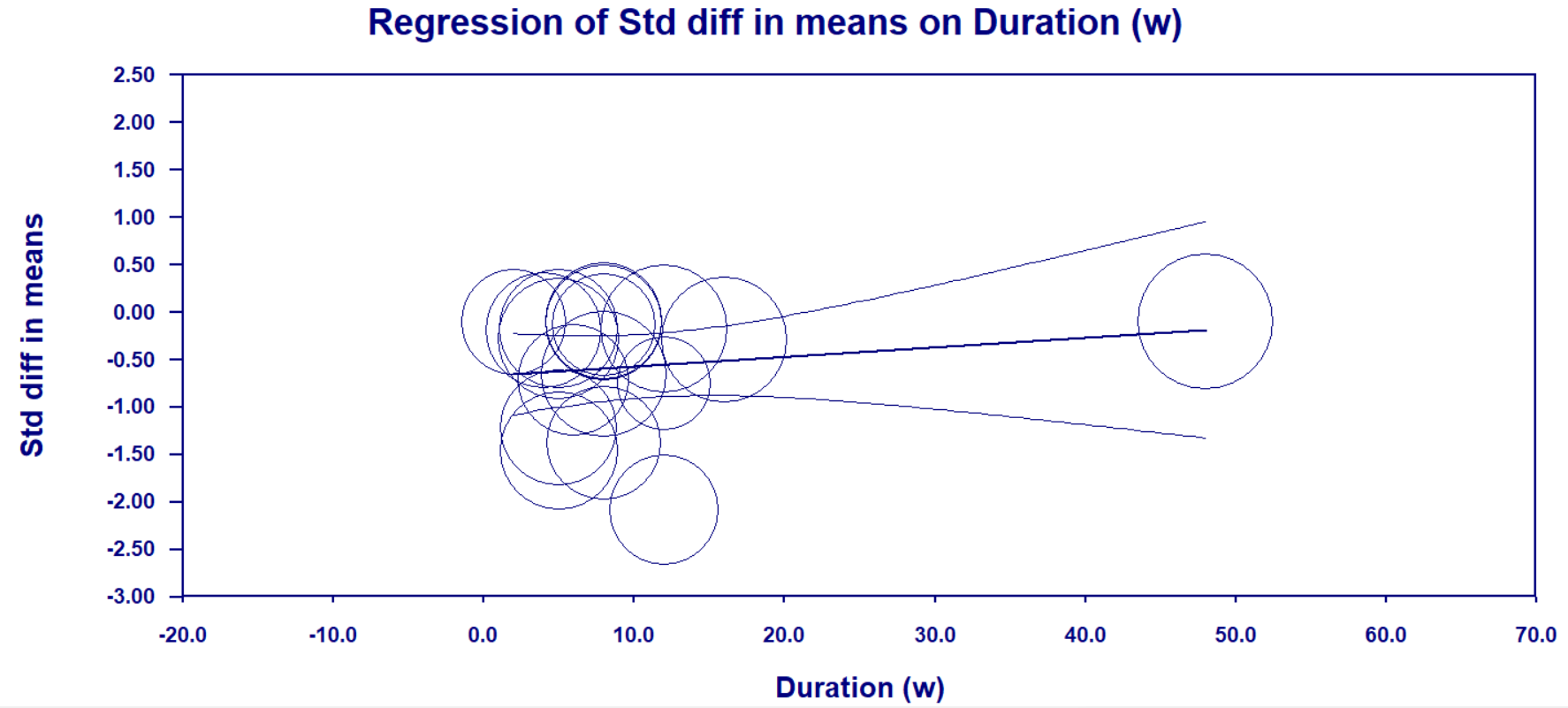


(b) Dose of serious games


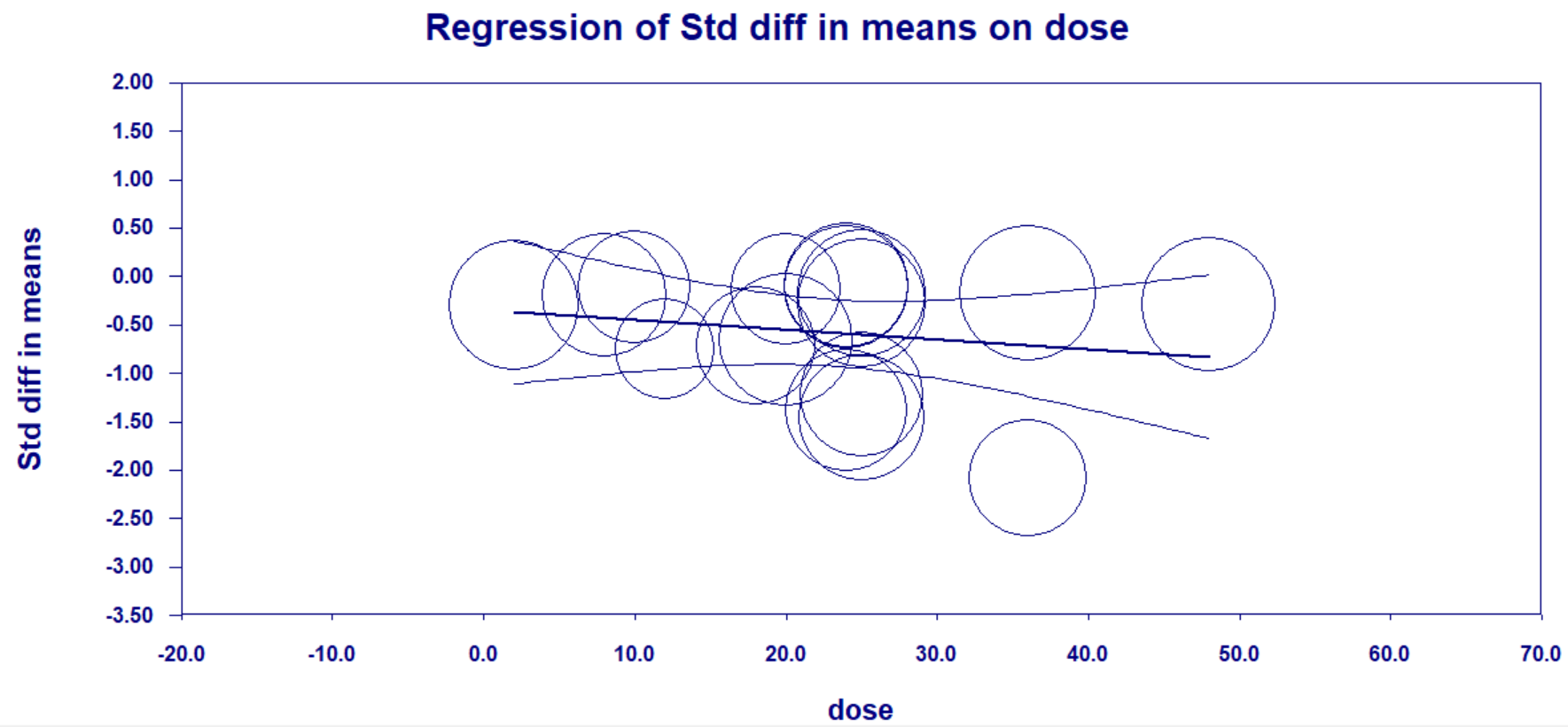

Supplement: Multimedia Appendix 3 [file jmir_v24i9e37753_app3.docx]
