# Supplementary figures and images for: Effects of Serious Games on Depression in Older Adults: Systematic Review and Meta-analysis of Randomized Controlled Trials
Source: J Med Internet Res. 2022 Sep 6;24(9):e37753. doi: 10.2196/37753 (PMC9490522; doi:10.2196/37753)

## Funnel plot of this study


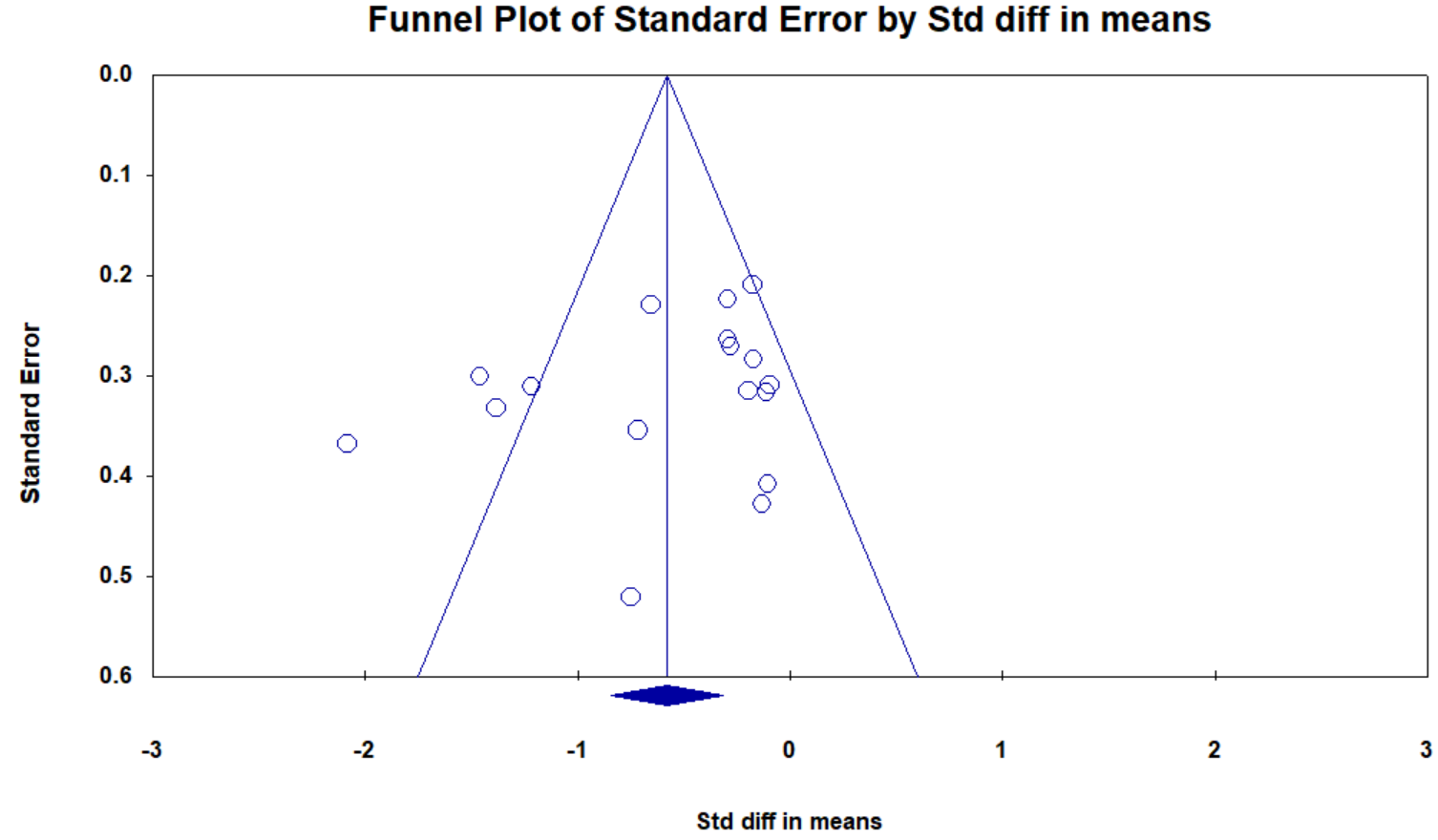

Supplement: Multimedia Appendix 4 [file jmir_v24i9e37753_app4.docx]
